# Evaluation of dehydrated corn silage as the primary forage for lactating dairy cows

**DOI:** 10.3168/jdsc.2022-0268

**Published:** 2022-09-30

**Authors:** S. Hisadomi, M. Oba

**Affiliations:** 1 Department of Agricultural, Food and Nutritional Science, University of Alberta, Edmonton, Alberta, Canada T6G 2P5

## Abstract

The objective was to compare productivity of lactating dairy cows fed dehydrated corn silage with those fed whole-crop barley silage. Twelve multiparous Holstein cows in mid lactation were fed diets containing dehydrated corn silage (DCS) or barley silage with additional grain (BSHG) or without (BSLG) in a 3 × 3 Latin square design, with 21-d periods, balanced for carryover effects. The dietary starch content was similar for DCS and BSHG diets, and dietary forage content was similar for DCS and BSLG diets. Experimental diets did not affect dry matter intake, but cows fed DCS diet decreased milk yield compared with those fed barley silage regardless of the dietary starch content. Apparent total-tract digestibility of starch and crude protein was also lower for cows fed DCS compared with those fed barley silage, and milk urea N content was lowest for cows fed DCS diet, indicating that DCS likely had less protein degradation in the rumen than barley silage. Milk fat content and yield, energy-corrected milk yield, and feed efficiency were not different between cows fed BSHG and DCS diets, but higher for cows fed BSLG than those fed BSHG or DCS diet, which can be attributed to the difference in dietary starch content. Feeding DCS in place of barley silage did not improve productivity of lactating dairy cows in the current study, and further research is warranted to optimize its utilization in dairy diets.

## Highlights

• Feeding barley silage in a low starch diet maximized feed efficiency of dairy cows.
• Feeding dehydrated corn silage did not affect feed intake.
• Dehydrated corn silage had lower digestibility of starch and protein.
• Milk yield was lower for cows fed dehydrated corn silage than barley silage.
• Feeding dehydrated corn silage did not affect production within a high starch diet.

## Graphical Abstract

**Figure:**
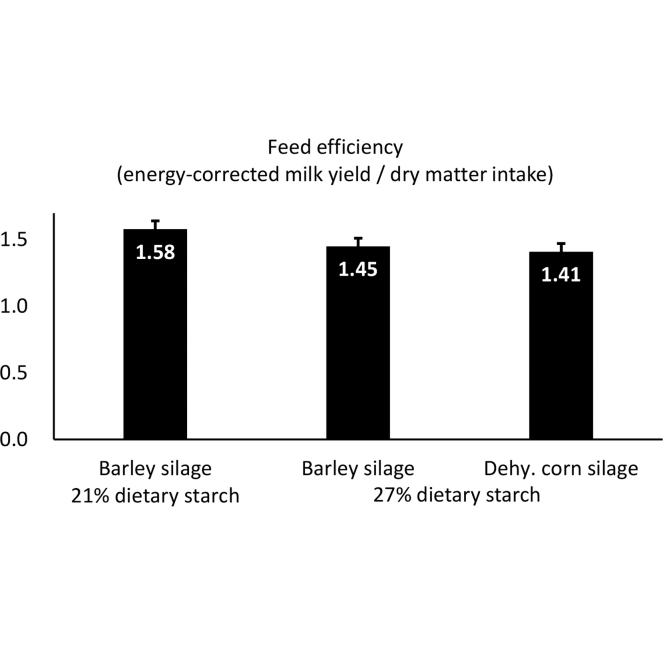
**Summary:** Feed efficiency, along with milk fat yield and fat-corrected milk yield, was highest for cows fed barley silage at 21% dietary starch, but they were similar between cows fed barley silage and dehydrated corn silage at 27% dietary starch. Feeding dehydrated corn silage in place of barley silage did not improve productivity of lactating dairy cows in the current study, and further research is warranted to optimize its utilization in dairy diets.

A substantial amount of dry forages are exported from the United States, Canada, and Australia to Asian countries where agricultural land for forage production is limited, and utilized as the primary forage in the diet of lactating dairy cows. In addition, dairy producers who usually grow their own forages may also use dry forages produced elsewhere when forage supply is in shortage due to drought or flooding. Hay is a common dry forage marketed internationally to minimize the transportation cost per unit of DM, but its energy content is relatively low due to its low starch content. Corn silage is the primary forage used in the diet of lactating dairy cows in many countries around the world, but its role in the international forage market is limited due to the high moisture content. Corn silage, which is high in starch content, can be dried and marketed as dehydrated corn silage (**DCS**) as a forage alternative. However, to our knowledge, little information exists about the feeding value of DCS. The objective of this research was to compare productivity of lactating dairy cows fed DCS with those fed whole-crop barley silage, a conventional forage used in the western Canadian dairy industry. We hypothesized, based on previous research that compared corn grain and barley grain (McCarthy et al., 1989; Overton et al., 1995; Silveira et al., 2007), that cows fed DCS would increase DMI and milk production.

All experimental procedures were pre-approved by the University of Alberta Animal Care and Use Committee for Livestock (AUP#3971). Twelve multiparous Holstein cows in mid lactation (732 ± 51.6 kg of BW, 116 ± 35.5 DIM, 2.64 ± 0.12 BCS; mean ± SD) were used for the study conducted at the Dairy Research and Technology Centre at the University of Alberta (Edmonton, AB, Canada). Whole-crop barley was harvested at mid-dough stage in July 2021 at a 15-mm theoretical length of cut (**TLC**) using a John Deere 8700 and 8800 (Deere and Company), and ensiled for at least 6 mo before the study. Whole-crop corn was harvested at 33% DM and 50% milk line in September 2020 at the 19-mm TLC with a kernel processor using a John Deere 7550 (Deere and Company). The whole-crop corn was ensiled for at least 15 mo, dried using a Limones LN-15.000 dryer (Industrial Limones-Gont S.L.U.) at 95 to 100°C for 20 to 25 min, and compressed to 40 cm × 60 cm × 35 cm bales (approximately 35 kg per bale) at Barr-Ag Ltd. (Olds; Table 1). Cows were fed diets containing DCS or barley silage with additional grain (**BSHG**) or without (**BSLG**) in a 3 × 3 Latin square design, with 21-d periods, balanced for carryover effects. The dietary starch content was similar for DCS and BSHG diets, and dietary forage content was similar for DCS and BSLG diets (Table 2). Water was added to TMR containing the DCS to make its DM content similar to BSLG. All experimental diets were formulated with AMTS Cattle Professional version 4.15.0 (Agricultural Modeling and Training Systems LLC) to meet or exceed the nutrient requirements of a 680-kg BW cow producing 41 kg/d of milk with 4.0% fat and 3.3% CP. All animals were housed in individual tiestalls and had free access to water. Cows were fed experimental diets as TMR once daily at 0730 h at 105 to 110% of actual feed intake of previous day. Cows were milked in their stalls twice daily at 0600 and 1700 h.

**Table 1:** Nutrient composition, in vitro digestibility, and particle size distribution of barley silage and dehydrated corn silage

Item	Barley silage	Dehydrated corn silage^1^
Nutrient composition		
DM, %	31.4	92.6
OM, %	92.3	94.4
CP, % DM	12.3	8.9
NDF, % DM	42.5	37.8
Starch, % DM	16.0	28.1
In vitro digestibility		
NDF 30 h, % NDF	58.9	60.3
Starch 7 h, % starch	85.8	75.7
Particle size distribution^2^		
>19.0 mm, %	8.2	2.1
19.0–8.0 mm, %	71.6	27.7
8.0–1.18 mm, %	19.5	51.3
<1.18 mm, %	0.7	18.9

1 Dehydrated corn silage was dried using a Limones LN-15.000 dryer (Industrial Limones-Gont S.L.U.) at 95 to 100°C for 20 to 25 min, and compressed to 40 cm × 60 cm × 35 cm bales (approximately 35 kg per bale) at Barr-Ag Ltd. (Olds).

2 Particle size distributions were measured by a Penn State Particle Separator.

**Table 2:** Ingredients, nutrient composition, and particle size distribution of experimental diets fed to lactating dairy cows^1^

Item	BSHG	BSLG	DCS
Ingredient, % DM			
Barley silage	41.2	51.3	—
Dehydrated corn silage	—	—	51.3
Dry ground corn grain	11.4	11.3	11.3
Dry ground barley grain	22.3	8.3	8.3
Canola meal	17.2	14.4	14.4
Bypass soybean meal^2^	1.5	7.3	7.3
Beet pulp	2.6	3.5	3.5
Mineral and vitamin mix^3^	2.0	1.9	1.9
Limestone	1.4	1.1	1.1
Commercial fat supplement^4^	0.3	0.7	0.7
Calcium phosphate	0.1	0.2	0.2
Nutrient composition			
DM, %	51.0	46.1	46.1
Forage NDF, % DM	17.8	22.1	19.9
NDF, % DM	28.2	31.0	28.8
CP, % DM	18.4	18.8	17.1
Starch, % DM	27.1	21.1	27.4
Particle size distribution^5^			
19.0 mm, %	2.9	3.7	0.8
19.0–8.0 mm, %	40.7	44.2	30.9
8.0–1.18 mm, %	36.2	34.9	65.1
<1.18 mm, %	20.2	17.2	3.2

1 BSHG = barley silage and high grain supplement mix diet; BSLG = barley silage and low grain supplement mix diet; DCS = dehydrated corn silage and low grain supplement mix diet.

2 Soyplus (Landus).

3 Mineral and vitamin mix contained 13.5% Na, 10.1% Ca, 8.9% Cl, 4.5% Mg, 2.4% P, 1.1% S, 0.3% K, 3,186 mg/kg Zn, 2,746 mg/kg Mn, 1,180 mg/kg Fe, 508 mg/kg Cu, 54 mg/kg Co, 45 mg/kg I, 14 mg/kg Se, 315 kIU vitamin A, 74 kIU vitamin D, 2,248 IU vitamin E.

4 Jefo dairy fat 99% (Jefo Nutrition Inc.).

5 Particle size distributions were measured by Penn State Particle Separator.

Individual DMI was recorded daily throughout the experiment. Forage and concentrate samples were collected 3 consecutive days at the end of each period and composited to yield one sample per period. The DM concentrations of forage and concentrate samples were determined in a forced-air oven at 55°C for 48 h. Experimental diets were adjusted with the determined DM as necessary to feed the same experimental diets on a DM basis. Particle size distribution of forage and experimental diets were measured by Penn State Particle Separator with 3 screens of 19.0, 8.0, and 1.18 mm and a pan described in Kononoff et al. (2003). Milk yield was recorded daily for all cows. Milk samples were collected from 4 consecutive milkings at the end of each period. Fecal samples were collected every 9 h during the last 3 d of each period (1300, 2200, 0700, 1600, 0100, 1000, 1900, and 0400 h) and composited to make one sample per cow per period accounting for diurnal variation. Fecal samples were dried in a forced-air oven at 55°C for 72 h.

Dried forage, concentration mixes and fecal samples were ground with a Wiley mill (Thomas Scientific) and analyzed by Cumberland Valley Analytical Services (Waynesboro, PA) for DM (AOAC International, 2002; method 930.15), CP (AOAC International, 2000; method 990.03), NDF (Van Soest et al., 1991), and starch (Hall, 2009). Indigestible NDF (**iNDF**), determined after 240 h of in vitro digestion, was used as an internal marker to estimate apparent total-tract nutrient digestibility (**ATTD**; Cochran et al., 1986). The ATTD was calculated with the following equation:

ATTD (% of nutrient intake) = 100 − [100 × (dietary iNDF content, %DM/fecal iNDF content, %DM) × (fecal nutrient content, %DM/dietary nutrient content, %DM)].

Milk samples were analyzed individually for concentrations of milk fat, milk CP, lactose, MUN, and SCC by mid-infrared spectroscopy (ISO-IDF, 2013; ISO 9622|IDF 141; Foss System MilkoScan 7RM, Foss North America) at the Lactanet Canada Central Milk Testing Laboratory (Edmonton, AB, Canada). The 4% FCM was calculated as (0.4 × milk yield (kg/d) + 15 × fat yield (kg/d), and ECM was calculated as (0.3246 × milk yield, kg) + (12.86 × milk fat yield, kg) + (7.04 × milk CP yield, kg) according to the equations described by Tyrrell and Reid (1965) and Bernard (1997), respectively. Feed efficiency was calculated as ECM divided by DMI.

Data from one cow were removed due to various health issues throughout the study. Statistical analyses were conducted using the Fit Model procedure of JMP (version 14, SAS Institute Inc.) with the following model:

Y_ijk_ = μ + T_i_ + P_j_ + C_k_ + e_ijk_,

where Y_ijk_ is the observation for dependent variables, μ is the overall mean, T_i_ is the fixed effect of treatment (i = 1 to 3), P_j_ is the fixed effect of period (j = 1 to 3), C_k_ is the random effect of cow (k = 1 to 11), and e_ijk_ is the residual error. When overall treatment effect was significant, Bonferroni *t*-test was used to separate treatment means. Significance was declared when *P* < 0.05 and tendencies were discussed when *P* < 0.10.

Experimental diets did not affect DMI, but cows fed DCS diet decreased milk yield (*P* < 0.01) compared with those fed barley silage regardless of the dietary forage content (Table 3). Milk fat content of DCS cows was not different from those fed barley silage, but it was lower for cows fed BSHG than those fed BSLG diet (*P* < 0.05). Yields of milk fat, 4% FCM, and feed efficiency (ECM/DMI) were higher for cows fed BSLG (*P* < 0.05), but not different between BSHG and DCS diets. Milk urea N content was lower for cows fed DCS compared with those fed barley silage (*P* < 0.05). Similarly, apparent total-tract digestibility of starch and CP was lower for cows fed DCS compared with those fed barley silage (*P* < 0.01).

**Table 3:** DMI, production performance, and apparent total-tract digestibility of cows fed experimental diets differing in primary forage^1^

Variable	LSM			SE	*P*-value
	BSHG	BSLG	DCS		
DMI, kg/d	32.3	30.9	32.4	1.11	0.26
Yield, kg/d					
Milk	45.2^a^	44.9^a^	42.2^b^	2.33	<0.01
Fat	1.61^b^	1.82^a^	1.61^b^	0.072	<0.01
CP	1.55	1.54	1.47	0.052	0.08
Lactose	2.03	2.03	1.95	0.103	0.07
4% FCM	42.1^b^	45.3^a^	41.4^b^	1.37	<0.01
ECM	46.2^ab^	48.8^a^	45.0^b^	1.40	<0.01
Composition					
Fat, %	3.72^b^	4.15^a^	3.84^ab^	0.249	0.02
CP, %	3.52	3.49	3.45	0.111	0.29
Lactose, %	4.52	4.52	4.54	0.0267	0.63
SCC, × 1,000/mL	55.9	55.7	65.2	19.5	0.74
MUN, mg/dL	14.1^a^	15.8^a^	11.8^b^	0.78	<0.0001
ECM/DMI	1.45^b^	1.58^a^	1.41^b^	0.062	<0.01
Apparent total-tract digestibility, %					
DM	70.9	68.0	68.6	0.85	0.09
CP	71.6^a^	69.0^a^	63.9^b^	1.22	<0.01
NDF	48.7	48.3	48.2	1.40	0.95
Starch	99.2^a^	99.1^a^	97.8^b^	0.22	<0.001

a–c Within a row, least squares means with different superscripts differ (*P* < 0.05).

1 BSHG = barley silage with high grain; BSLG = barley silage with low grain; DCS = dehydrated corn silage with low grain.

Ruminal starch digestibility is generally lower for corn grain than barley grain (Allen, 2000). However, in previous studies, reduced ruminal starch digestion for corn grain was associated with greater DMI, leading to greater energy intake and greater milk production (McCarthy et al., 1989; Overton et al., 1995; Silveira et al., 2007). Reduced starch digestion can increase DMI because of less propionate flux, which decreases metabolic satiety signal sent from the liver (Allen, 2000). However, in the current study, feeding DCS, which is lower in starch digestibility, did not increase DMI. It is speculated that metabolic satiety from propionate metabolism may not have been dominant in feed intake regulation under the current experimental conditions.

In the current study, ATTD of CP was also lower for cows fed DCS compared with those fed barley silage. Forages provided 27.5, 33.6, and 26.7% of total dietary CP, respectively, for BSHG, BSLG, and DCS diets, and it is possible that differences in CP digestibility of forages affected milk production. Our findings are consistent with previous reports; ATTD was slightly lower (Khorasani et al., 2001) or tended to be lower (McCarthy et al., 1989; Overton et al., 1995) for diets containing corn grain in place of barley grain. As such, low CP digestibility can be at least partly attributed to forage type (e.g., corn silage vs. barley silage), but the possibility that the dehydration process affected protein degradability should not be excluded. Application of high temperature can denature protein and decrease its digestibility (Böttger and Südekum, 2018). Although drying time was relatively short (20–25 min), heat application (95–100°C) to dehydrate corn silage may have decreased its CP degradability. Consistent with protein digestibility data, MUN content was lowest for cows fed DCS diet, even though their starch digestion in the rumen is expected to be lower, indicating that DCS likely reduced CP degradation in the rumen.

Enhanced production of milk fat and 4% FCM, and greater feed efficiency for cows fed BSLG diet can be explained by the difference in dietary starch content rather than the difference in forage type; dietary starch content was similar between DCS and BSHG diets, whereas it was approximately 6 percentage units lower for the BSLG diet. Although ruminal pH was not measured in the current study, it is possible that feeding a low starch diet (i.e., BSLG diet) prevented ruminal pH depression, reducing the accumulation of CLA, which inhibit fatty acid synthesis in the mammary gland (Bauman and Griinari, 2003), and increasing milk fat production.

Although feeding DCS did not improve productivity of dairy cows in the current study, the results should be interpreted with caution because we were not able to identify the specific mechanism by which the DCS diet decreased milk production. The current study did not evaluate the corn silage before dehydration as a control, and we could not isolate specific effects of the dehydration process. Digestibility of DCS may have been affected by the dehydration process, but it may simply be due to differences in forage type or growing condition of forages used in the current study. It should be noted that feed efficiency was similar between DCS and BSHG diets and that we did not evaluate DCS at different dietary allocations or dietary starch contents. As observed in cows fed barley silage (i.e., BSHG vs. BSLG), animal responses to DCS may be affected by dietary starch content. In addition, potential deficiencies of DCS can be compensated by diet formulation approaches; for example, DCS can be fed with forages that are high in RDP to provide sufficient MP. The current study provided preliminary information about DCS, but further research is warranted to optimize its utilization.
